# A Radiobrominated Tyrosine Kinase Inhibitor for EGFR with L858R/T790M Mutations in Lung Carcinoma

**DOI:** 10.3390/ph14030256

**Published:** 2021-03-12

**Authors:** Muammar Fawwaz, Kenji Mishiro, Ryuichi Nishii, Akira Makino, Yasushi Kiyono, Kazuhiro Shiba, Seigo Kinuya, Kazuma Ogawa

**Affiliations:** 1 Graduate School of Medical Sciences, Kanazawa University, Kakuma-machi, Kanazawa, Ishikawa 920-1192, Japan; muammar.fawwaz@umi.ac.id; 2 Faculty of Pharmacy, Universitas Muslim Indonesia, Urip Sumoharjo KM. 10, Makassar 90-231, Indonesia; 3 Institute for Frontier Science Initiative, Kanazawa University, Kakuma-machi, Kanazawa, Ishikawa 920-1192, Japan; mishiro@p.kanazawa-u.ac.jp; 4 National Institute of Radiological Sciences (NIRS), QST, Inage-ku, Chiba 263-8555, Japan; nishii.ryuichi@qst.go.jp; 5 Biomedical Imaging Research Center (BIRC), University of Fukui, Eiheiji-cho, Yoshida-gun, Fukui 910-1193, Japan; amakino@u-fukui.ac.jp (A.M.); ykiyono@u-fukui.ac.jp (Y.K.); 6 Advanced Science Research Center, Kanazawa University, Takara-machi, Kanazawa, Ishikawa 920-8640, Japan; shiba@med.kanazawa-u.ac.jp; 7 Department of Nuclear Medicine, Institute of Medical, Pharmaceutical and Health Sciences, Kanazawa University, Takara-machi, Kanazawa, Ishikawa 920-8641, Japan; kinuya@med.kanazawa-u.ac.jp

**Keywords:** tyrosine kinase inhibitors, L858R/T790M, imaging probes, bromine-labeled, PET

## Abstract

Activating double mutations L858R/T790M in the epidermal growth factor receptor (EGFR) region are often observed as the cause of resistance to tyrosine kinase inhibitors (TKIs). Third-generation EGFR-TKIs, such as osimertinib and rociletinib (CO-1686), was developed to target such resistance mutations. The detection of activating L858R/T790M mutations is necessary to select sensitive patients for therapy. Hence, we aimed to develop novel radiobromine-labeled CO-1686 as a positron emission tomography (PET) imaging probe for detecting EGFR L858R/T790M mutations. Nonradioactive brominated-CO1686 (BrCO1686) was synthesized by the condensation of *N*-(3-[{2-chloro-5-(trifluoromethyl)pyrimidin-4-yl}amino]-5-bromophenyl) acrylamide with the corresponding substituted 1-(4-[4-amino-3-methoxyphenyl]piperazine-1-yl)ethan-1-one. The radiobrominated [^77^Br]BrCO1686 was prepared through bromodestannylation of the corresponding tributylstannylated precursor with [^77^Br]bromide and *N-*chlorosuccinimide. Although we aimed to provide a novel PET imaging probe, ^77^Br was used as an alternative radionuclide for ^76^Br. We fundamentally evaluated the potency of [^77^Br]BrCO1686 as a molecular probe for detecting EGFR L858R/T790M using human non-small-cell lung cancer (NSCLC) cell lines: H1975 (EGFR L858R/T790M), H3255 (EGFR L858R), and H441 (wild-type EGFR). The BrCO1686 showed high cytotoxicity toward H1975 (IC_50_ 0.18 ± 0.06 µM) comparable to that of CO-1686 (IC_50_ 0.14 ± 0.05 µM). In cell uptake experiments, the level of accumulation of [^77^Br]BrCO1686 in H1975 was significantly higher than those in H3255 and H441 upon 4 h of incubation. The radioactivity of [^77^Br]BrCO1686 (136.3% dose/mg protein) was significantly reduced to 56.9% dose/mg protein by the pretreatment with an excess CO-1686. These results indicate that the binding site of the radiotracers should be identical to that of CO-1686. The in vivo accumulation of radioactivity of [^77^Br]BrCO1686 in H1975 tumor (4.51 ± 0.17) was higher than that in H441 tumor (3.71 ± 0.13) 1 h postinjection. Our results suggested that [^77^Br]BrCO1686 has specificity toward NSCLC cells with double mutations EGFR L858R/T790M compared to those in EGFR L858R and wild-type EGFR. However, the in vivo accumulation of radioactivity in the targeted tumor needs to be optimized by structural modification.

## 1. Introduction

Overexpression of epidermal growth factor receptor (EGFR) and mutation in the tyrosine kinase (TK) domain have been shown to be highly associated with a variety of tumors, including non-small-cell lung cancer (NSCLC) [1]. Activation of EGFR-TK is involved in the proliferation, metastasis, angiogenesis, invasion, and suppression of apoptosis [2]. Therefore, EGFR is an attractive target for tumor imaging and therapy [3]. As the therapeutic effect of EGFR-TK inhibitors (TKIs) depends on EGFR mutation status, it is important to determine this status before administrating them [3]. Imaging agents for determining EGFR status could serve as an alternative strategy to predict the drug efficacy instead of gene diagnosis with repeated invasive biopsy [4,5].

EGFR-TKIs are effective molecular targeted drugs for NSCLC patients with L858R mutation [6,7]. However, almost all patients acquired resistance to the first-generation EGFR-TKIs, such as gefitinib and erlotinib [8]. A secondary EGFR mutation in codon 790 (threonine-790 to methionine) of exon 20 of this gene has been reported in approximately 50% of all cases that develop resistance to EGFR-TKI therapy [6]. Crystal structure analysis showed that T790M hinders the interaction of EGFR with the first-generation EGFR-TKIs [7]. This mutation is believed to be induced by the treatment with first-generation TKIs because it is rarely detected in untreated patients [8]. Thus, to overcome the resistance, the third-generation EGFR-TKIs possessing a high-affinity against EGFR with L858R/T790M double mutations were developed. At the same time, companion diagnosis is also important for the third-generation EGFR-TKIs. We recently synthesized and evaluated a radiotracer, [^125^I]ICO1686, which is a derivative of one of the third-generation EGFR-TKIs, CO-1686, with a high affinity for EGFR with L858R/T790M double mutations. [^125^I]ICO1686 could detect the EGFR with L858R/T790M double mutations in vitro; however, it showed insufficient tumor uptake and low tumor/blood ratio of radioactivity [9].

Radiobromine has some advantages over other radiohalogens. For example, ^76^Br is a promising radionuclide for positron emission tomography (PET) with a relatively long half-life (16.2 h). It makes it possible to deliver the radiopharmaceuticals to distant sites and to take delayed PET imaging, such as at one day postinjection, which is impossible for ^18^F-labeled probes. Meanwhile, bromine has chemical properties similar to those of iodine. Therefore, for the synthesis of a radiobromine-labeled probe, a synthetic scheme for a radioiodine-labeled probe can be applied without substantial modification. Moreover, the smaller size of bromine than for iodine may not hinder the binding site [10]. Hence, we aimed to provide a novel radiobromine-labeled CO-1686 to determine the EGFR L858R/T790M mutation for the selection of patients sensitive to third-generation EGFR-TKIs.

**Scheme 1:**
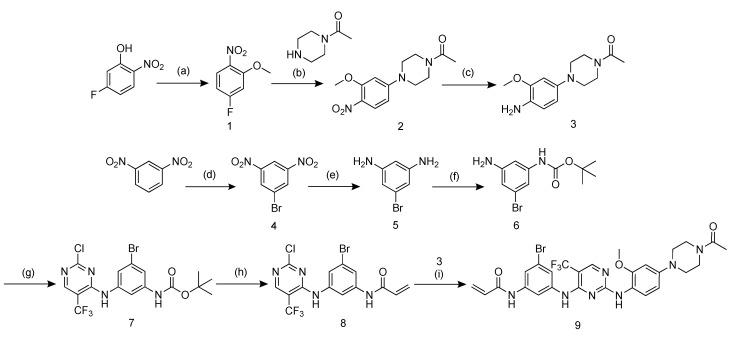
Synthesis of non-radioactive brominated CO-1686. (a) K_2_CO_3_, CH_3_I, acetone, 0 °C, 5 h; (b) DIPEA, DMA, 90 °C, overnight; (c) Pd/C 10%, H_2_, MeOH, rt, 5 h; (d) H_2_SO_4_, NBS, 80 °C, 1.5 h; (e) SnCl_2_·2H_2_O, ethanol, rt, 3 h; (f) Boc_2_O, MeCN, 50 °C, overnight; (g) 2,4-dichloro-5(trifluoromethyl) pyrimidine, DIPEA, *n*-butanol, 0 °C, 2 h, rt, 4 h; (h-1) HCl/ethyl acetate, rt, 1 h; (h-2) acryloyl chloride, DIPEA, dichloromethane, −30 °C to rt, 2 h; (i) TFA, 1,4-dioxane, 60 °C, 3 h.

**Scheme 2:**
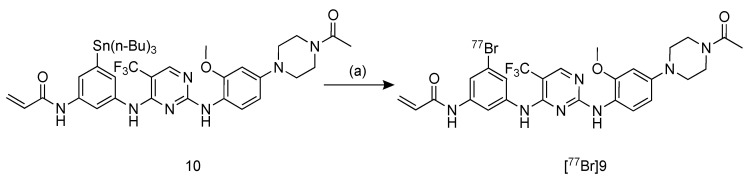
Radiosynthesis of compound [^77^Br]**9**. (a) [^77^Br]Br^–^, acetic acid, NCS, ethanol, 80 °C, 60 min.

In this study, a non-radioactive brominated *N*-(3-{[2-({4-[4-acetylpiperazin-1-yl]-2-methoxyphenyl}amino)-5- (trifluoromethyl)pyrimidin-4-yl]amino}-5-bromophenyl)acrylamide (BrCO1686, Scheme 1, **9**) and a radioactive brominated **9** ([^77^Br]BrCO1686, Scheme 2, [^77^Br]**9**) were synthesized and evaluated in vitro and in vivo using three different human NSCLC cell lines; H1975 (EGFR with L858R/T790M dual mutations), H3255 (EGFR with L858R mutation), and H441 (wild-type EGFR). Although we are interested in developing ^76^Br-labeled probes for PET, in these initial studies, ^77^Br was used because of its longer half-life (57.0 h).

## 2. Results

### 2.1. Probe Design and Synthesis

A derivative of CO-1686, brominated compound **9**, was prepared by multistep synthesis starting from commercially available 1,3-dinitrobenzene (Scheme 1). A bromine atom was introduced in the diaminophenyl group of CO-1686 using *N*-bromosuccinimide (NBS). The bromine was attached in this substituent because this would not substantially influence the affinity of CO-1686 toward EGFR L858R/T790M, according to the complex crystal structure of CO-1686 and T790M EGFR [11]. The most important substituents playing a pivotal role in the binding of CO-1686 to EGFR L858R/T790M are the anilinopyrimidine core, methoxy, and trifluoromethyl groups [11,12].

### 2.2. Cytotoxicity Assays

The half-maximal inhibitory concentration (IC_50_) of **9** was determined by 2-(2-methoxy-4-nitrophenyl)-3-(4-nitrophenyl)-5-(2,4-disulfophenyl)-2H tetrazolium monosodium salt (WST-8) assay. Our previous study showed that iodinated CO-1686 (ICO1686) has a similar cytotoxicity effect with parent compound CO-1686 toward mutated NSCLC. In this study, the cytotoxicity of **9** toward H1975 and H3255 was similar to those of ICO1686 and CO-1686, as shown in Table 1. This suggests that the bromine substituent on **9** did not significantly influence the activity toward mutated EGFR, in terms of both active mutant L858R EGFR and double mutations L858R/T790M EGFR. In contrast, the IC_50_ of **9** toward H441 was significantly lower than that of ICO1686 (*p* = 0.0178). The data were statistically analyzed and presented as mean ± standard deviation (SD).

### 2.3. Radiosynthesis of [^77^Br]BrCO1686 ([^77^Br]9)

The [^77^Br]**9** was synthesized by a bromodestannylation reaction with the corresponding tributyltin precursor **10**, as shown in Scheme 2. This radiotracer was synthesized using *N*-chlorosuccinimide (NCS) as an oxidizing agent in an acidic solution at 80 °C in moderate radiochemical yield (45%). After purification using reversed phase-high-performance liquid chromatography (RP-HPLC), the radiochemical purity was over 99%. The identity of [^77^Br]**9** was confirmed by comparing its retention time with that of nonradioactive **9** in the HPLC analyses (Figure S1). These analyses showed that the retention time of [^77^Br]**9** (9.13 min) was slightly shorter than that of [^125^I]ICO1686 (11.76 min).

### 2.4. Determination of Partition Coefficient

The log *p* value for [^77^Br]**9** was 1.72 ± 0.21, which indicates that [^77^Br]**9** has appropriate lipophilicity for passive membrane penetration. The log *p* value of [^77^Br]**9** was significantly lower than that of [^125^I]ICO1686, which was 1.84 ± 0.01 (*p* = 0.0097) [9]. This result is consistent with the reverse-phase HPLC analysis, in which the retention time of [^77^Br]**9** was shorter than that of [^125^I]ICO1686.

### 2.5. Stability Assessment

The stability profile of [^77^Br]**9** in phosphate-buffered saline (PBS) and murine plasma is presented in Supporting Information (Figure S2). [^77^Br]**9** was >80% intact in PBS and murine plasma after incubation for 1 h at 37 °C. Its purity decreased to 57% after 24 h of incubation.

### 2.6. Cellular Uptake Studies

**Figure 1:**
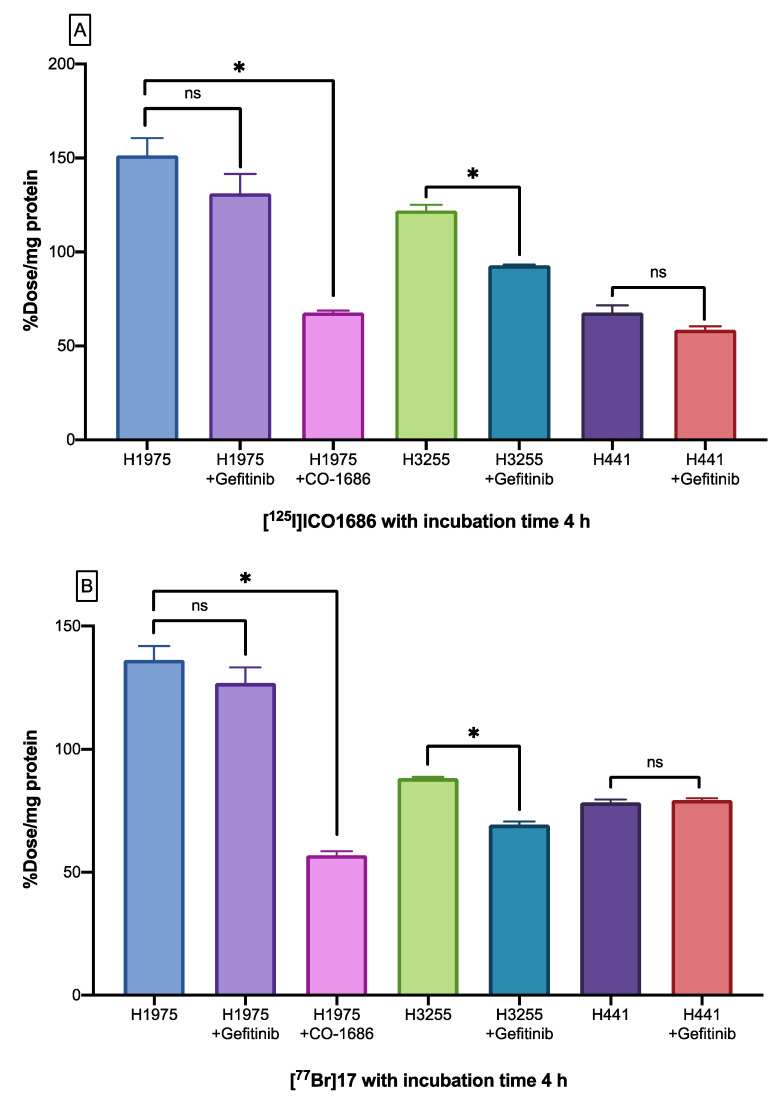
The cell uptake of (**A**) [^125^I]ICO1686 and (**B**) [^77^Br]**9** in H1975, H3255, and H441 cell lines after 4 h incubation. Significance was determined using Dunnett’s multiple-comparison test or unpaired Student’s *t*-test (* *p* < 0.05) ns: not significant.

Data on the radioactivity uptake are expressed as percent dose per milligram protein (% dose/mg protein), as shown in Figure 1. [^77^Br]**9** and [^125^I]ICO1686 exhibited preferential accumulation in H1975 (L858R/T790M double mutations EGFR) upon 4 h of incubation, which was significantly higher than that observed with H3255 (L858R active mutant EGFR) and H441 (wild-type EGFR). In the in vitro blocking experiment toward H1975 cells, the accumulation of radioactivity of [^77^Br]**9** (136.3% dose/mg protein) and [^125^I]ICO1686 (151.3% dose/mg protein) was reduced to 56.9 and 67.7% dose/mg protein, respectively, by the pretreatment with an excess CO-1686 (*p* < 0.0001 and *p* < 0.0001, respectively). Meanwhile, pretreatment with excess gefitinib toward H1975 was meaningless but significantly reduced the accumulation of both radiotracers in H3255 cells.

### 2.7. Biodistribution Studies

The biodistributions of [^77^Br]**9** and [^125^I]ICO1686 were studied in normal mice, as summarized in Table 2. These data are expressed as percent injected dose per gram (% ID/g). At 4 h after injection, the distribution pattern in normal organs was similar among the groups, with the highest uptake in the intestine and liver. The accumulation of [^77^Br]**9** in blood was much higher than that of [^125^I]ICO1686. The accumulation of both radiotracers in mice bearing H1975 and H441 tumors is summarized in Table 3. The accumulation of [^77^Br]**9** in H1975 tumor at 1 h postinjection was higher than that of [^125^I]ICO1686. As shown in Table 4, regarding the biodistribution of [^77^Br]bromide ion in normal mice, it was retained for a long time in the blood and some organs.

## 3. Discussion

The modulation of EGFR expression has been used as a target for therapy and diagnosis due to its highly specific targeting capability. The therapeutic effects of EGFR-TKIs in NSCLC can be assessed by PET imaging of EGFR, which can provide more accurate information to aid in the selection of patients for individualized therapy [13]. In recent years, several PET probes based on EGFR-TKI derivatives for detecting EGFR mutation and/or predicting treatment response in NSCLC have been reported [13].

In our previous study, an in vitro kinase inhibition assay with ICO1686 and CO-1686 showed their high selectivity toward double mutations EGFR L858R/T790M compared with that toward wild-type EGFR [9]. Therefore, we conducted a further study by designing and synthesizing radiobrominated [^77^Br]**9** as a surrogate of [^76^Br]**9**, which can be used as a molecular imaging agent for EGFR L858R/T790M because bromine could be a bioisostere of iodine. A bromine atom was introduced in the diaminophenyl group of CO-1686, as was done with the iodine derivative in the previous study. As expected, introducing bromine at this position did not significantly affect the CO-1686 affinity toward EGFR L858R/T790M because it does not play a key role in the binding activity of CO-1686 to EGFR L858R/T790M based on the complex crystal structure analysis [11]. As a result, the IC_50_ of **9** was similar to that of the iodinated compounds ICO1686 and CO-1686.

Concerning ^77^Br labeling, radiobromination was achieved in the presence of an oxidizing agent NCS with a moderate radiochemical yield of approximately 45%. Radiobromine was directly introduced to the aromatic group by a destannylation reaction with a positively charged radiobromine generated in situ [14].

Cellular uptake studies of both the radiotracers showed a significant difference among the three cells. Preferential accumulation of [^77^Br]**9** and [^125^I]ICO1686 was seen in H1975, which has EGFR double mutations. In the in vitro blocking studies, both the radiotracers showed specificity in H1975 cells. The methoxyl group contributes to the specificity of CO-1686 toward EGFR, and the trifluoromethyl substituent of CO-1686 is beneficial for forming hydrophobic interactions with Met790, which is not afforded by a wild-type gatekeeper residue with Thr790 [11]. The specificity of both the radiotracers toward H1975 cells was evaluated by two independent experiments using two blocking agents, CO-1686 and gefitinib. As expected, the uptake of [^77^Br]**9** and [^125^I]ICO1686 in H1975 was significantly reduced by almost 60% upon pretreatment with CO-1686, which is known to bind to EGFR-TK with L858R/T790M double mutations. In contrast, the uptake of [^77^Br]**9** and [^125^I]ICO1686 in H1975 was not significantly reduced with gefitinib, which is known to be ineffective to EGFR-TK with L858R/T790M double mutations. These results indicate that the radiotracers bound to EGFR-TK with L858R/T790M double mutations compete with CO-1686. Moreover, gefitinib only reduced the radioactivity uptake of radiotracers in H3255. Although H1975 cells carry the EGFR L858R mutation, the presence of secondary mutation T790M interferes with the affinity of first-generation TKIs, such as gefitinib, to the ATP binding site [15,16].

In the biodistribution study, [^77^Br]**9** and [^125^I]ICO1686 were co-injected into the mice to minimize the number and increase the reliability of the data in the biodistribution study. At 4 h postinjection, the distribution patterns in normal organs were similar among the groups, with the highest uptake in the intestine and liver. High uptake in the liver and small intestine indicated that radiotracers were mainly eliminated through hepatobiliary clearance because both radiotracers have relatively hydrophobic structures. However, the clearance rate of [^77^Br]**9** was slow from the blood and other organs. The [^125^I]ICO1686 was eliminated at a rate of up to 71% in feces, while only 35% of [^77^Br]**9** was eliminated at 24 h postinjection. The high radioactivity of radiobromine-labeled tracers in the blood is strongly related to the free bromide ions [17,18,19]. Therefore, the radioactivity in the blood can be used as an index of the debromination of radiobromine-labeled compounds in vivo [20]. In this study, the accumulation of [^77^Br]**9** in the blood was much higher than that of [^125^I]ICO1686, which suggested that debromination occurred in vivo. Our experiment on the biodistribution of [^77^Br]bromide showed long-term retention in the blood and some organs (Table 4). In this context, we confirmed that radioactivity in the blood after the injection of [^77^Br]**9** should be caused by free bromide ions from debrominated [^77^Br]**9**. Debromination has also been observed with some radiotracers, such as 4-[^76^Br]-bromo-α-methyl-L-phenylalanine (4-[^76^Br]BAMP) and 3-[^76^Br]-bromo-α-methyl-L-tyrosine ([^76^Br]BAMT) [14,21].

In biodistribution studies using tumor-bearing mice, the accumulation of [^77^Br]**9** was higher than that of [^125^I]ICO1686 in H1975 tumors (Table 3). According to a biodistribution study of [^77^Br]bromide, the blood clearance of [^77^Br]bromide was slow, and free bromide was distributed throughout the whole body (Table 4). The accumulation of free radiobromide generated by the debromination of [^77^Br]**9** would contribute to its high uptake in the tumor and all organs. Thus, we assume that after injection of [^77^Br]**9**, the specificity to tumors with EGFR L858R/T790M mutations should be low. Therefore, it is desirable to develop more stable and selective analogs that highly accumulate in target tumors.

Previous studies have suggested that the high and rapid hepatobiliary excretion of radiotracers must have been derived from the high lipophilicity, which may hinder the accumulation of these radiotracers in the targeted tumor [22,23]. Some studies have shown that the introduction of a short-chain PEG in radiotracers improved pharmacokinetics and accumulation in the tumor, presumably because the hepatobiliary excretion rate was reduced due to the increased hydrophilicity [12,23,24]. Therefore, we suppose that the structural modification of [^77^Br]**9**, such as PEG introduction, might improve its tumor accumulation.

## 4. Material and Methods

### 4.1. General Chemistry

Solvents and reagents were purchased from Nacalai Tesque, Inc. (Kyoto, Japan), Tokyo Chemical Industry, Co., Ltd. (Tokyo, Japan), Fujifilm Wako Pure Chemical Corporation (Osaka, Japan), Kanto Chemical, Co., Inc. (Tokyo, Japan), Merck (Darmstadt, Germany), and Chemescene (Monmouth Junction, NJ, USA), and used as provided. CO-1686 was purchased from AdooQ BioScience (Irvine, Canada). Gefitinib was purchased from Fujifilm Wako Pure Chemical Corporation (Osaka, Japan). Thin-layer chromatography (TLC) was performed on silica plates 60 F_254_ (Merck, Darmstadt, Germany).

Nuclear magnetic resonance (NMR) spectra were recorded on a JNM-ECS400 (400 MHz) or JNM-ECA600 (600 MHz) spectrometer (JEOL Ltd., Tokyo, Japan). The mass spectrometry was performed on a JMS-T700 (JEOL Ltd., Tokyo, Japan) on fast atom bombardment mass spectrometry (FAB-MS). HPLC analyses and purification were carried out on a Shimadzu Prominence HPLC system (Shimadzu Corp., Kyoto, Japan). The optical density in WST-8 assays were measured on an Infinite^®^ F200 Pro microplate reader (TECAN, Männedorf, Switzerland). Radioactivity was determined using an auto gamma counter ARC-7010B (Hitachi, Ltd., Tokyo, Japan).

### 4.2. Probe Design and Synthesis

Design and synthesis procedures were shown in Scheme 1 and Scheme 2. Intermediate compounds were synthesized according to the previous studies, with a slight modification [9,25,26,27,28]. For compounds **1**–**3** and tin precursor **10**, materials synthesized in the previous study [9] were used. NMR spectra are provided in the Supporting Information (Figures S3–S8).

#### 4.2.1. Synthesis of 1-bromo-3,5-dinitrobenzene (**4**)

Compound **4** (7.0 g, 70%) was synthesized according to the previous report [28]. ^1^H NMR (400 MHz, CDCl_3_): δ 8.72 (2H, d, *J =* 1.6 Hz), 9.01 (1H, t, *J =* 1.6 Hz). ^13^C NMR (100 MHz, CDCl_3_): δ 117.7, 123.8, 132.2, 149.1. HRMS (FAB+) calculated for C_6_H_3_BrN_2_O_4_ [M + H]^+^: *m/z* = 245.9298, found 245.9276.

#### 4.2.2. Synthesis of 5-bromobenzene-1,3-diamine (**5**)

Compound **5** (5.26 g, 99%) was synthesized according to the previous report [29]. ^1^H NMR (400 MHz, CDCl_3_): δ 3.60 (4H, br s), 5.91 (1H, s), 6.25 (2H, d, *J* = 2.0 Hz). ^13^C NMR (100 MHz, CDCl_3_): δ 100.1, 108.8, 123.7, 148.5. HRMS (FAB+) calculated for C_6_H_7_BrN_2_ [M + H]^+^: *m/z* = 185.9777, found 185.9793.

#### 4.2.3. Synthesis of tert-butyl (3-amino-5-bromophenyl)carbamate (**6**)

To a stirred mixture of **5** (5.26 g, 28.13 mmol, 1.0 eq.) and acetonitrile (50 mL), di-tert-butyl dicarbonate (Boc_2_O) (6.14 g, 28.13 mmol, 1 eq.) was added at 50 °C and the mixture was stirred overnight under nitrogen atmosphere. After the reaction was completed, the solvent was removed by evaporation. The crude product was purified by column chromatography on silica gel (hexane/ethyl acetate = 7/3) and concentrated under reduced pressure to afford **6** (4.45 g, 55%) as a crystal orange. ^1^H NMR (400 MHz, CDCl_3_): δ 1.51 (9H, s), 3.73 (2H, s), 6.42 (1H, s), 6.50 (1H, s), 6.79 (2H, s). ^13^C NMR (100 MHz, CDCl_3_): δ 28.2, 80.8, 103.3, 111.3, 112.6, 123.1, 140.5, 148.3, 152.5. HRMS (FAB+) calculated for C_11_H_15_BrN_2_O_2_ [M + H]^+^: *m/z* = 286.0300, found 286.0317.

#### 4.2.4. Synthesis of tert-butyl(3-{[2-chloro-5-(trifluoromethyl)pyrimidin-4-yl]amino}-5- bromophenyl)carbamate (**7**)

To a stirred mixture of **6** (4.45 g, 15.50 mmol, 1.0 eq.) and *n*-butanol (31 mL), 2,4-dichloro-5(trifluoromethyl) pyrimidine (2 mL, 1 eq., purchased from Chemscene), *N*,*N*-diisopropylethylamine (DIPEA) (5 mL, 2 eq.) were added at 0 °C and stirred for 2 h. The stirring was continued at room temperature for 4 h. After the reaction was completed, the solvent was removed by evaporation. The crude product was purified using column chromatography on silica gel (hexane/ethyl acetate = 7/3) and concentrated under reduced pressure to afford **7** (4.1 g, 85%) as pale-yellow solid**.** ^1^H NMR (400 MHz, CDCl_3_): δ 1.54 (9H, s), 6.59 (1H, s), 7.00 (1H, s), 7.39 (1H, s), 7.48 (1H, s), 7.66 (1H, s), 8.46 (1H, s). ^13^C NMR (100 MHz, CDCl_3_): δ 28.2, 81.7, 106.8 (q, *J_CF_* = 30.5 Hz), 110.6, 118.6, 119.6, 122.8, 123.4 (q, *J_CF_* = 271.8 Hz), 137.8, 140.2, 152.3, 156.1 (q, *J_CF_* = 4.8 Hz), 157.1, 163.6. HRMS (FAB+) calculated for C_16_H_15_BrClF_3_N_4_O_2_ [M + H]^+^: *m/z* = 465.9854, found 465.9842.

#### 4.2.5. Synthesis of *N*-(3-{[2-chloro-5-(trifluoromethyl)pyrimidin-4-yl]amino}-5- bromophenyl)acrylamide (**8**)

A mixture of **7** (4.1 g, 8.77 mmol, 1.0 eq.) and 4.0 M hydrochloric acid (HCl)/ethyl acetate (1.0 mL) was stirred at room temperature for 1 h. After completion of the reaction, the mixture was concentrated under reduced pressure to give a corresponding de-Boc product as a colorless solid. The crude product was used for the next reaction without further purification. To a stirred mixture of the crude product (3.0 g) and DIPEA (4.3 mL, 24.48 mmol, 3 eq.) in dichloromethane (81 mL), acryloyl chloride (1 mL, 12.24 mmol, 1.5 eq.) was added at −30 °C and warmed to room temperature. After stirring for 2 h, the crude product was diluted with ethyl acetate, washed with 1 M HCl, saturated aqueous NaHCO_3_, and brine successively. The crude product was purified using column chromatography on silica gel (dichloromethane/methanol = 200/1), crystalized from chloroform/hexane to afford **8** (200 mg, 10%) as a white solid. ^1^H NMR (400 MHz, CDCl_3_): δ 5.84 (2H, d, *J* = 7.2 Hz), 6.24 (1H, dd, *J* = 11.2, 6.8 Hz), 6.47 (1H, d, *J* = 11.2 Hz), 7.43 (1H, s), 7.50 (1H, s), 7.66 (1H, s), 7.98 (1H, s), 8.63 (1H, s). ^13^C NMR (100 MHz, (CD_3_)_2_SO): δ 101.3 (q, *J_CF_* = 70.5 Hz), 109.8, 116.2, 117.6, 121.6, 123.9 (q, *J_CF_* = 270.8 Hz), 127.8, 131.8, 140.8, 141.5, 157.0 (q, *J_CF_* = 4.8 Hz), 161.3, 163.7, 166.2. HRMS (FAB+) calculated for C_14_H_9_BrClF_3_N_4_O [M + H]^+^: *m/z* = 419.9702, found 419.9600.

#### 4.2.6. Synthesis of *N*-(3-{[2-({4-[4-acetylpiperazin-1-yl]-2-methoxyphenyl}amino)-5- (trifluoromethyl)pyrimidin-4-yl]amino}-5-bromophenyl)acrylamide (**9**)

To a stirred mixture of compound **8** (120 mg, 0.28 mmol, 1.0 eq.) and 2 M trifluoroacetic acid (TFA) in dioxane (0.6 mL), compound **3** (70 mg, 0.28 mmol, 1 eq.) was added at room temperature. The mixture was warmed to 60 °C and stirred for 3 h. After completion of the reaction, the pH of the reaction mixture was adjusted to neutral (7–8) by the addition of saturated aqueous NaHCO_3_, and the mixture was extracted with ethyl acetate. The organic phase was separated, dried over anhydrous Na_2_SO_4_, filtered, and concentrated under reduced pressure. The crude product was purified by an isocratic HPLC system equipped with a Cosmosil^®^ 5SL-II (20 ID × 250 mm) column (Nacalai Tesque) with a mobile phase of ethyl acetate/methanol (97/3) at a flow rate of 9.5 mL/min. The column temperature was maintained at 40 °C. The product was concentrated under reduced pressure to afford **9** (80 mg, 45%) as a white solid. ^1^H NMR (400 MHz, CDCl_3_): δ 2.16 (3H, s), 3.12–3.20 (4H, m), 3.64 (2H, t, *J* = 4.4 Hz), 3.79 (2H, t, *J* = 4.8 Hz), 3.91 (3H, s), 5.80 (1H, d, *J* = 10.8 Hz), 6.21 (1H, dd, *J* = 15.2, 10.4 Hz), 6.44 (1H, d, *J* = 15.6 Hz), 6.54–6.60 (2H, m), 7.11 (1H, s), 7.25 (1H, s), 7.43 (1H, s), 7.56 (1H, s), 7.66 (1H, s), 7.74 (1H, s), 8.07 (1H, d, *J* = 8.8 Hz), 8.30 (1H, s). ^13^C NMR (100 MHz, (CD_3_)_2_SO): δ 16.9, 41.2, 47.3, 51.0, 51.5, 60.2, 113.0 (q, *J_CF_* = 40.5 Hz), 116.2, 124.5, 127.4, 135.3, 136.9, 138.9, 142.1, 146.4 (q, *J_CF_* = 334.9 Hz), 147.1, 149.6, 154.8, 165.5, 167.1, 177.1, 180.9, 184.2 (q, *J_CF_* = 6.0 Hz), 186.8, 191.2, 194.4, 200.7. HRMS (FAB+) calculated for C_27_H_27_BrF_3_N_7_O_3_ [M + H]^+^: *m/z* = 633.1324, found 633.1311.

### 4.3. Cytotoxicity Assays

WST-8 assay was used to evaluate IC_50_ of **9** toward NSCLC cell lines (H1975, H441 and H3255) as described previously [9]. The reference compounds; CO-1686, ICO-1686, and gefitinib, have been evaluated in our previous study using the same source of cell lines [9].

### 4.4. Production of Bromine-77

^77^Br was produced by ^77^Se(p,n)^77^Br reaction with and separated according to a previous study at the University of Fukui [20,30].

### 4.5. Radiosynthesis of [^77^Br]BrCO1686 ([^77^Br]**9**)

Radiotracers, [^77^Br]**9** was synthesized by a bromodestannylation reaction of the corresponding precursor **10** (Scheme 1). A solution of [^77^Br]bromide (2.5 MBq, 1 μL) was charged into a sealed vial containing **10** (1 mg/mL, 10 μL) in ethanol, acetic acid (5%, 30 μL), and NCS (20 mg/mL, 10 μL). After vortexed for 5 min, the mixture was shaken at 80 °C for 60 min. The reaction was quenched by aqueous sodium hydrogen sulfite (5 mg/mL, 10 μL), and the reaction solution was purified by a gradient HPLC system equipped with a Cosmosil^®^ 5C_18_ MS-II (4.6 ID × 150 mm) column (Nacalai Tesque) with a varying mobile phase (water/methanol = 30/70–0/100 in 20 min) at a flow rate of 1 mL/min. The column temperature was maintained at 40 °C. The [^77^Br]**9** was isolated after confirmation by comparing the retention times of the radiobrominated and nonradioactive **9** in the HPLC analysis. Radioactivity was determined by an auto well gamma counter.

### 4.6. Determination of Partition Coefficient

To determine the lipophilicity, the partition coefficient of [^77^Br]**9** was measured as described previously [31]. The partition coefficient (log *P*) was expressed as the ratio of radioactivity in *n*-octanol and radioactivity in 0.1 M phosphate buffer (pH 7.4).

### 4.7. Stability Assessment

To confirm the stability, we measured the purity of radiotracer after incubation in PBS and mouse plasma, respectively, as described previously [9].

### 4.8. Cellular Uptake Studies

H1975 (1 × 10^5^ cells/well), H441 (1 × 10^5^ cells/well), and H3255 (2 × 10^5^ cells/well) NSCLC cells were cultured in 6 well plates. Cells were incubated in medium with 10% FBS and 100 IU/mL penicillin–streptomycin at 37 °C in a humidified atmosphere with 5% CO_2_ for 24 h. The cell culture was then incubated in an FBS-free medium containing [^125^I]ICO1686, which was prepared by the method reported previously [9] and [^77^Br]**9** (3.7 kBq/well, respectively) for 4 h, and then washed with 1 mL of ice-cold PBS. The cells were dissolved by a 1.0 M NaOH aqueous solution (0.5 mL × 2). The cell lysate was transferred into a test tube. The radioactivity of ^77^Br was measured by an auto well gamma counter at a 100–600 keV energy range. The crossover of ^125^I activity into the ^77^Br channel was negligible. After one month of experiments, the radioactivity of ^125^I was measured at a 16–71 keV energy range because the radioactivity of ^77^Br was negligible at the time. In the blocking assays, CO-1686 or gefitinib (100 µM, respectively) was co-incubated with the radiotracer, and the cellular uptake was assessed using the same method as mentioned above. The BCA protein assay kit protocol was used to determine the protein concentration.

### 4.9. Tumor Model

Six weeks old male ddY mice and four weeks old female BALB/c nu/nu mice were purchased from Japan SLC Inc. (Hamamatsu, Japan). Tumor-bearing mice were prepared by subcutaneous injection of cells suspended in 100 μL medium to the female BALB/c nu/nu mice. H1975 cells (5 × 10^6^ cells) were implanted in the right shoulder, and H441 cells (3 × 10^6^ cells) were implanted in the left shoulder. The tumor reached palpable size after post-inoculation 12 and 9 days for H1975 and H441, respectively.

### 4.10. Biodistribution Studies

Mice were injected intravenously with double tracers [^77^Br]**9** (10 kBq) and [^125^I]ICO1686 (25 kBq) in 100 μL of saline solution containing 1% tween-80 and 10% ethanol via the lateral tail vein. The ddY mice were sacrificed by decapitation at 10 min, 1, 4, and 24 h. Meanwhile, the tumor-bearing mice were sacrificed at 1 h and 6 h postinjection (4 mice/group). The selected tissues and organs were collected, weighed, and counted the radioactivity of ^77^Br using an auto well gamma counter. One month later, ^125^I activity was determined. The biodistribution data were expressed as% ID/g along with the SD. The same procedures were applied to perform the biodistribution study of [^77^Br]bromide ion (111 kBq).

### 4.11. Statistical Analysis

Statistical methods, one-way ANOVA followed by a Dunnett’s multiple-comparison test for cellular uptake studies in H1975 cells. Unpaired Student’s *t*-test was used to confirm the significances in the cytotoxicity assays, determination of partition coefficient, and the cellular uptake studies toward H3255 and H441 cells. All values are given as mean ± SD, and *p* < 0.05 was considered statistically significant.

## 5. Conclusions

Radiobrominated [^77^Br]**9** was successfully synthesized with high radiochemical purity. This radiotracer showed in vitro specificity toward NSCLC cells with EGFR L858R/T790M double mutations compared with that in EGFR L858R and wild-type EGFR. However, the in vivo accumulation in the target tumor was insufficient to visualize the tumor. Therefore, it is desirable to develop more stable and selective analogs that are rapidly cleared from the body and highly accumulate in the target tumor. The structural modification by introducing PEG could be an alternative to improve the tumor uptake of [^77^Br]**9**.

## Figures and Tables

**Table 1:** The IC_50_ of **9**, ICO1686, CO-1686, and gefitinib on NSCLC cell lines by WST-8 assay.

Cells Lines	Mutation Status	IC_50_ (μM)			
		9	ICO1686 *	CO-1686 *	Gefitinib *
H1975	L858R/T790M	0.18 ± 0.06	0.20 ± 0.05	0.14 ± 0.05	>10
H3255	L858R	0.20 ± 0.01	0.50 ± 0.21	0.15 ± 0.02	0.02 ± 0.02
H441	Wild type	0.64 ± 0.04	1.84 ± 0.44	0.26 ± 0.04	>10

Data represent the mean ± SD of three separate experiments. * Data from reference [9].

**Table 2:** Biodistribution of [^77^Br]**9** and [^125^I]ICO1686 at 10 min, 1, 4, and 24 h after i.v. injection in ddY mice.

Tissues	Time after Injection							
	10 min		1 h		4 h		24 h	
[^77^Br]**9**								
Blood	3.57	(0.21)	3.24	(0.30)	3.19	(0.18)	1.99	(0.28)
Liver	11.04	(0.55)	3.83	(0.38)	3.72	(0.30)	1.04	(0.21)
Kidney	11.68	(0.94)	4.21	(0.67)	2.81	(1.17)	1.33	(0.25)
Small intestine	25.38	(5.45)	26.49	(2.77)	5.23	(1.06)	1.00	(0.15)
Large intestine	2.83	(0.57)	3.18	(0.19)	47.73	(7.88)	0.98	(0.11)
Spleen	5.11	(3.46)	4.83	(1.82)	2.92	(2.02	1.14	(0.46)
Pancreas	3.78	(2.53)	3.58	(0.10)	3.04	(0.28)	1.13	(0.09)
Lung	8.72	(1.25)	4.97	(1.97)	5.08	(0.28)	1.62	(0.22)
Heart	4.57	(0.89)	2.93	(0.24)	1.87	(1.25)	0.80	(0.21)
Stomach ^‡^	6.17	(1.45)	7.00	(0.58)	6.68	(1.02)	2.21	(0.33)
Bone	2.76	(0.83)	1.66	(1.18)	1.54	(0.62)	0.89	(0.93)
Muscle	3.18	(0.55)	2.02	(0.55)	1.13	(0.78)	0.47	(0.20)
Brain	0.45	(0.30)	0.95	(0.14)	0.74	(0.50)	0.42	(0.09)
Urine	-		-		-		13.48	(1.28)
Feces	-		-		-		35.01	(6.85)
[^125^I]ICO1686								
Blood	1.41	(0.38)	0.17	(0.02)	0.06	(0.01)	0.13	(0.06)
Liver	19.21	(2.04)	7.40	(1.01)	6.45	(1.21)	1.40	(0.17)
Kidney	9.66	(1.19)	2.40	(0.56)	0.75	(0.18)	0.54	(0.10)
Small intestine	36.32	(12.00)	40.99	(4.13)	6.08	(1.99)	0.30	(0.11)
Large intestine	0.94	(0.18)	1.65	(0.34)	81.54	(10.19)	0.52	(0.55)
Spleen	2.73	(0.56)	0.55	(0.09)	0.20	(0.07)	0.13	(0.04)
Pancreas	2.03	(0.20)	0.86	(0.15)	0.12	(0.04)	0.05	(0.01)
Lung	3.61	(0.40)	0.59	(0.25)	0.16	(0.03)	0.14	(0.02)
Heart	2.24	(0.49)	0.30	(0.08)	0.08	(0.04)	0.20	(0.25)
Stomach ^‡^	2.60	(1.23)	1.64	(0.71)	0.76	(0.61)	0.05	(0.02)
Bone	0.74	(0.19)	0.16	(0.03)	0.05	(0.08)	0.09	(0.05)
Muscle	1.20	(0.14)	0.23	(0.09)	0.04	(0.01)	0.04	(0.02)
Brain	0.08	(0.01)	0.01	(0.01)	0.01	(0.00)	0.00	(0.00)
Urine ^‡^	-		-		-		1.63	(0.24)
Feces ^‡^	-		-		-		71.54	(6.53)

Data presented as % ID/g tissue. Each value represents mean ± SD for four mice. ^‡^ Presented as % ID/organ.

**Table 3:** Biodistribution of double tracers [^77^Br]**9** and [^125^I]ICO1686 at 1 and 6 h after i.v. injection in tumor-bearing mice.

Tissues	Time after Injection							
	1 h		6 h		1 h		6 h	
	[^77^Br]**9**				[^125^I]ICO1686			
Blood	5.64	(0.19)	5.35	(0.99)	0.50	(0.20)	0.17	(0.03)
Liver	15.89	(0.72)	6.76	(1.78)	30.32	(2.78)	10.16	(3.50)
Kidney	7.75	(0.85)	4.91	(0.80)	2.64	(1.45)	1.05	(0.41)
Small intestine	64.52	(3.35)	12.46	(5.93)	86.29	(2.99)	11.72	(8.01)
Large intestine	24.33	(17.81)	59.40	(18.22)	27.65	(21.75)	95.22	(32.67)
Spleen	9.19	(2.12)	2.04	(2.36)	1.80	(0.75)	0.25	(0.07)
Pancreas	12.95	(5.14)	4.28	(0.91)	2.71	(0.87)	0.36	(0.22)
Lung	11.42	(1.52)	6.29	(0.52)	1.62	(0.67)	0.37	(0.15)
Heart	5.36	(1.07)	2.99	(1.05)	0.61	(0.20)	0.53	(0.86)
Stomach ^‡^	1.31	(0.39)	1.27	(0.89)	1.49	(0.52)	1.17	(1.68)
Bone	1.93	(1.51)	1.95	(2.50)	0.42	(0.16)	0.22	(0.15)
Muscle	3.42	(1.19)	1.79	(0.30)	0.36	(0.18)	0.10	(0.05)
Brain	1.49	(0.16)	1.39	(0.17)	0.04	(0.01)	0.02	(0.03)
H1975	4.51	(0.17)	3.48	(0.50)	0.68	(0.11)	0.10	(0.02)
H441	3.71	(0.13)	3.30	(0.54)	0.44	(0.06)	0.19	(0.06)

Data presented as % ID/g tissue. Each value represents mean ± SD for four mice. ^‡^ Presented as % ID/organ.

**Table 4:** Biodistribution of [^77^Br]bromide at 15 min, 1, 4, and 24 h after i.v. injection in ddY mice.

Tissues	Time after Injection							
	15 min		1 h		4 h		24 h	
Blood	6.96	(0.58)	6.98	(0.45)	6.42	(0.64)	3.48	(0.36)
Liver	2.45	(0.29)	2.61	(0.17)	2.35	(0.30)	1.18	(0.12)
Kidney	5.10	(0.83)	4.58	(0.21)	4.51	(0.58)	2.62	(0.34)
Small intestine	3.94	(0.24)	3.92	(0.16)	3.18	(0.29)	1.99	(0.22)
Large intestine	3.12	(0.44)	2.78	(0.22)	2.37	(0.30)	1.55	(0.16)
Spleen	4.74	(0.31)	4.30	(0.26)	3.75	(0.73)	2.25	(0.36)
Pancreas	5.09	(0.78)	4.38	(0.27)	3.80	(0.37)	2.22	(0.32)
Lung	8.02	(0.95)	7.12	(0.48)	5.81	(0.59)	3.62	(0.32)
Heart	3.72	(0.47)	3.23	(0.09)	2.41	(0.20)	1.50	(0.19)
Stomach ^‡^	3.94	(0.54)	4.59	(1.18)	5.44	(1.23)	4.18	(1.00)
Bone	3.72	(0.44)	3.25	(0.24)	3.00	(0.38)	1.78	(0.30)
Muscle	2.28	(0.18)	1.92	(0.11)	1.63	(0.14)	0.98	(0.04)
Brain	1.08	(0.13)	1.49	(0.07)	1.45	(0.14)	0.84	(0.09)
Neck ^‡^	2.45	(0.50)	3.02	(0.35)	2.46	(0.42)	1.27	(0.28)
Urine ^‡^	-		-		-		20.74	(4.69)
Feces ^‡^	-		-		-		1.33	(0.49)

Data presented as % ID/g tissue. Each value represents mean ± SD for four mice. ^‡^ Presented as % ID/organ.

## Data Availability

The data presented in this study are available on request from the corresponding author.

## Supplementary Materials

The followings are available online at https://www.mdpi.com/1424-8247/14/3/256/s1, Figure S1: The chromatograms of (**a**) nonradioactive brominated compound **9** (BrCO1686) and (**b**) radioactive compound [*77*Br]**9** ([^77^Br]BrCO1686), Figure S2: The stability of radiolabeled compound [*77*Br]**9** in PBS and plasma, Figure S3: The NMR spectra of 1-bromo-3,5-dinitrobenzene (**4**), Figure S4: The NMR spectra of 5-bromobenzene-1,3-diamine (**5**), Figure S5: The NMR spectra of tert-butyl (3-amino-5-bromophenyl)carbamate (**6**), Figure S6: The NMR spectra of tert-butyl(3-{[2-chloro-5-(trifluoromethyl)pyrimidin-4-yl]amino}-5-bromophenyl)carbamate (**7**), Figure S7: The NMR spectra of *N*-(3-{[2-chloro-5-(trifluoromethyl)pyrimidin-4-yl]amino}-5- bromophenyl)acrylamide (**8**), Figure S8: The NMR spectra of *N*-(3-{[2-({4-[4-acetylpiperazin-1-yl]-2-methoxyphenyl}amino)-5-(trifluoromethyl)pyrimidin-4-yl]amino}-5-bromophenyl)acrylamide (**9**).
