# Supplementary material for: A Radiobrominated Tyrosine Kinase Inhibitor for EGFR with L858R/T790M Mutations in Lung Carcinoma
Source: Pharmaceuticals (Basel). 2021 Mar 12;14(3):256. doi: 10.3390/ph14030256 (PMC7998589; doi:10.3390/ph14030256)
Supplement: Supplementary file 1 [file pharmaceuticals-14-00256-s001.pdf]

Supporting Information

A Radiobrominated Tyrosine Kinase Inhibitor for EGFR with L858R/T790M Mutations in Lung Carcinoma

Muammar Fawwaz [1,2], Kenji Mishiro [3], Ryuichi Nishii [4], Akira Makino [5], Yasushi Kiyono [5], Kazuhiro Shiba [6], Seigo Kinuya [7], Kazuma Ogawa [1,3,*]

[1] Graduate School of Medical Sciences, Kanazawa University, Kakuma-machi, Kanazawa, Ishikawa 920-1192, Japan; muammar.fawwaz@umi.ac.id (M.F.)
[2] Faculty of Pharmacy, Universitas Muslim Indonesia, Urip Sumoharjo KM. 10, Makassar 90-231, Indonesia.
[3] Institute for Frontier Science Initiative, Kanazawa University, Kakuma-machi, Kanazawa, Ishikawa 920-1192, Japan; mishiro@p.kanazawa-u.ac.jp (K.M)
[4] National Institute of Radiological Sciences (NIRS), QST, Inage-ku, Chiba, Chiba 263-8555, Japan; nishii.ryuichi@qst.go.jp (R.N)
[5] Biomedical Imaging Research Center (BIRC), University of Fukui, Eiheiji-cho, Yoshida-gun, Fukui 910-1193, Japan; amakino@u-fukui.ac.jp (A.M); ykiyono@u-fukui.ac.jp (Y.K.)
[6] Advanced Science Research Center, Kanazawa University, Takara-machi, Kanazawa, Ishikawa 920-8640, Japan; shiba@med.kanazawa-u.ac.jp (K.S.)
[7] Department of Nuclear Medicine, Institute of Medical, Pharmaceutical and Health Sciences, Kanazawa University, Takara-machi, Kanazawa, Ishikawa 920-8641, Japan; kinuya@med.kanazawa-u.ac.jp (S.K.)

***Corresponding Author**

Institute for Frontier Science Initiative; Kanazawa University; Kakuma- machi, Kanazawa 920-1192; Japan.

Telephone/Fax: +81-76-234-4460

E-mail: kogawa@p.kanazawa-u.ac.jp

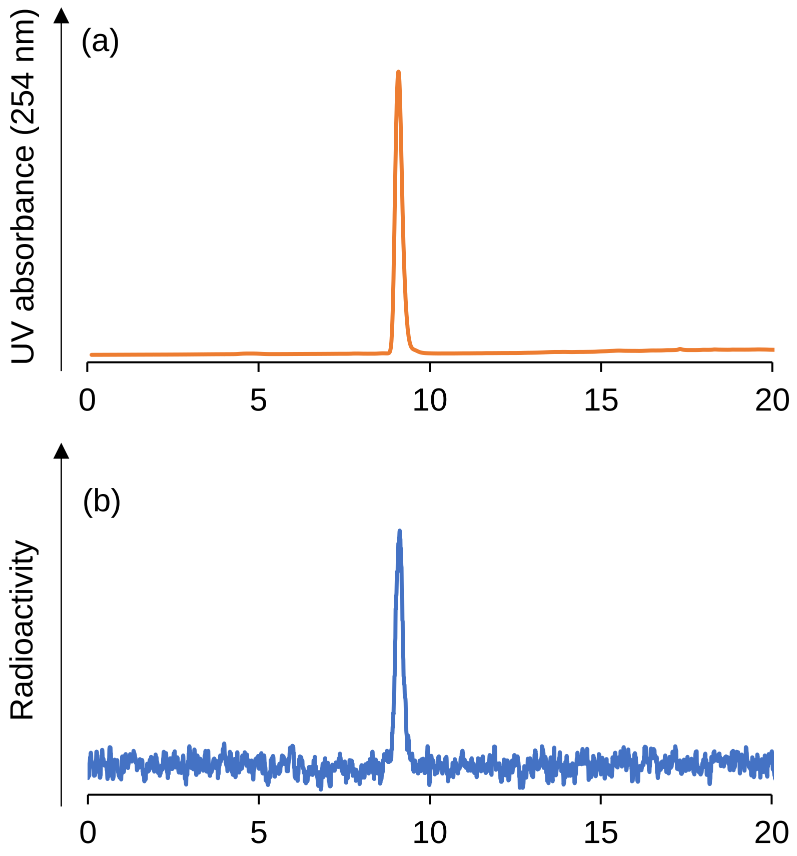

Figure S1. The chromatograms of (a) nonradioactive brominated compound **9** (BrCO1686) and (b) radioactive compound [^{77}Br]**9** ([^{77}Br]BrCO1686).

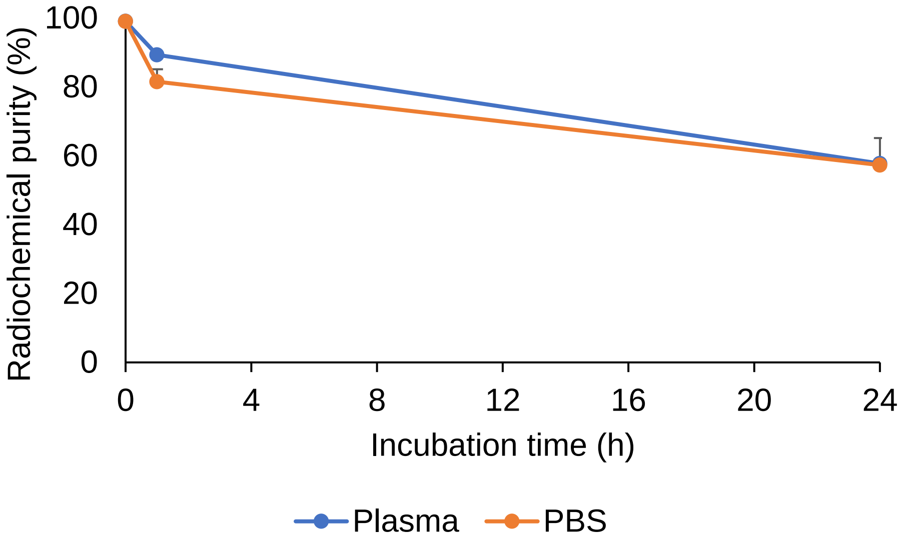

Figure S2. The stability of radiolabeled compound [^{77}Br]**9** in PBS and plasma.

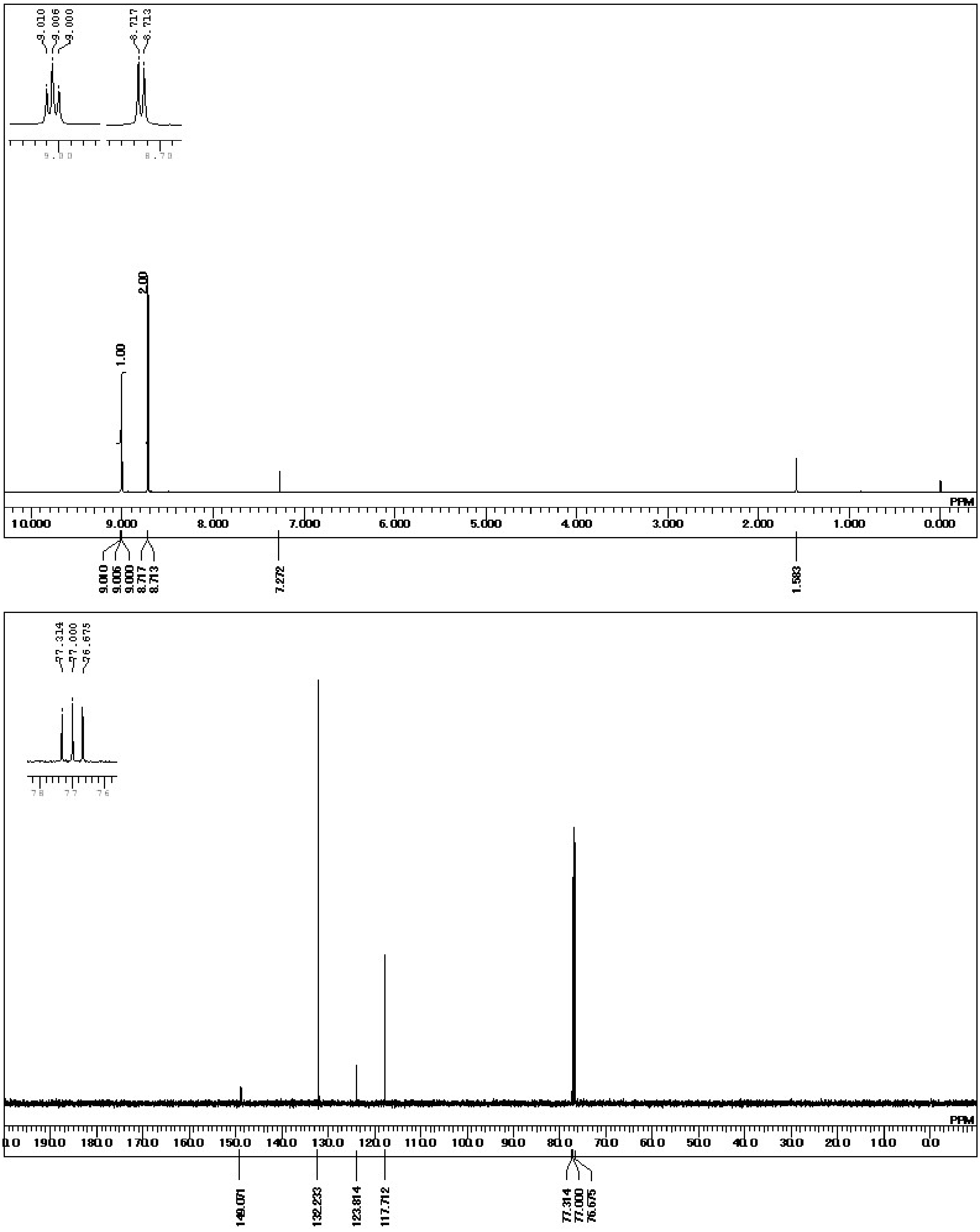

Figure S3. The NMR spectra of 1-bromo-3,5-dinitrobenzene (**4**). ^{1}H NMR (400 MHz, $CDCl_3$): δ 8.72 (2H, d, *J* = 1.6 Hz), 9.01 (1H, t, *J* = 1.6 Hz). ^{13}C NMR (100 MHz, $CDCl_3$): δ 117.7, 123.8, 132.2, 149.1. HRMS (FAB+) calculated for $C_6H_3BrN_2O_4$ $[M+ H]^+$: *m/z* = 245.9298, found 245.9276.

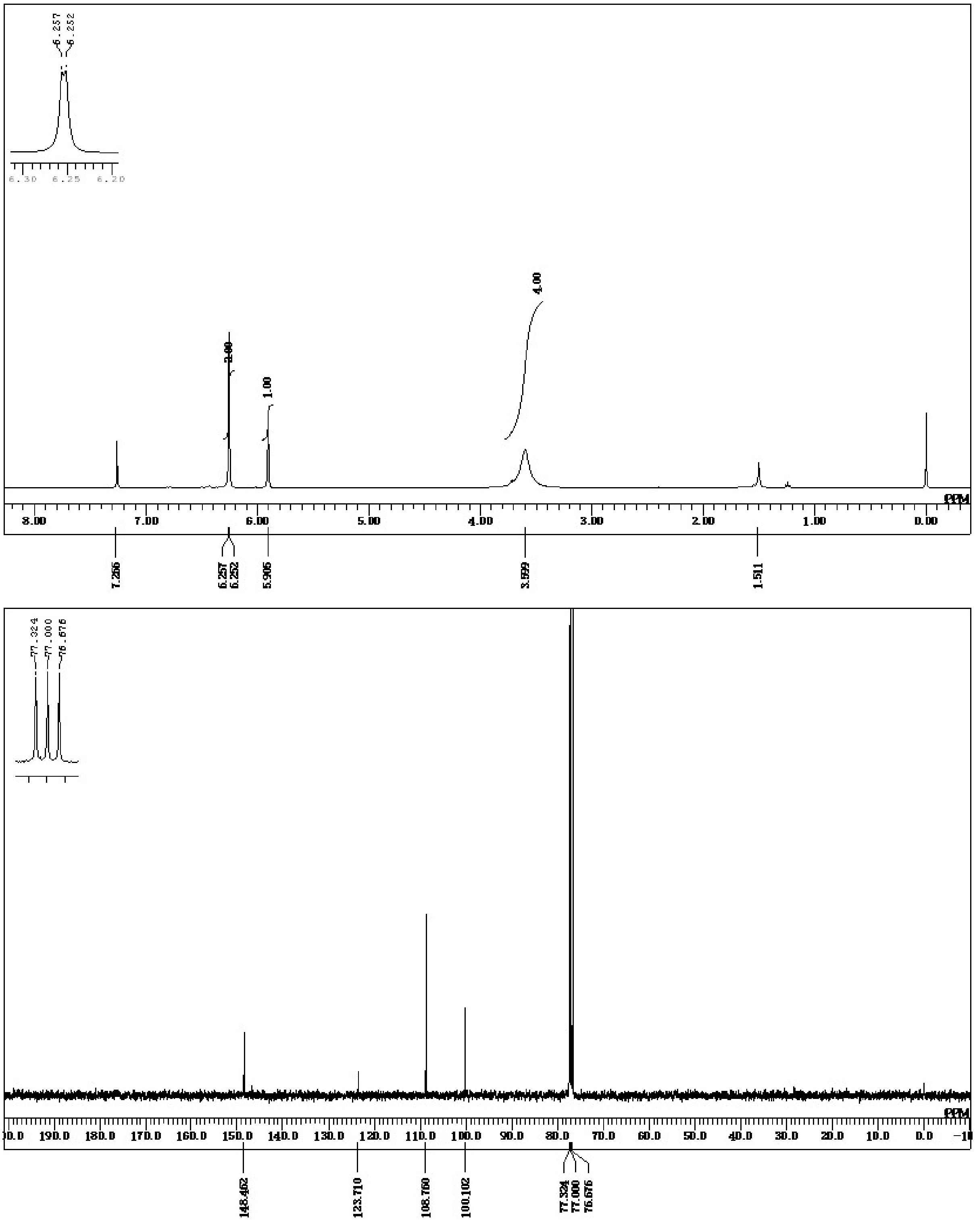

Figure S4. The NMR spectra of 5-bromobenzene-1,3-diamine (**5**). ^{1}H NMR (400 MHz, $CDCl_3$): δ 3.60 (4H, br s), 5.91 (1H, s), 6.25 (2H, d, J = 2.0 Hz). ^{13}C NMR (100 MHz, $CDCl_3$): δ 100.1, 108.8, 123.7, 148.5. HRMS (FAB+) calculated for $C_6H_7BrN_2$ $[M+H]^+$: m/z = 185.9777, found 185.9793.

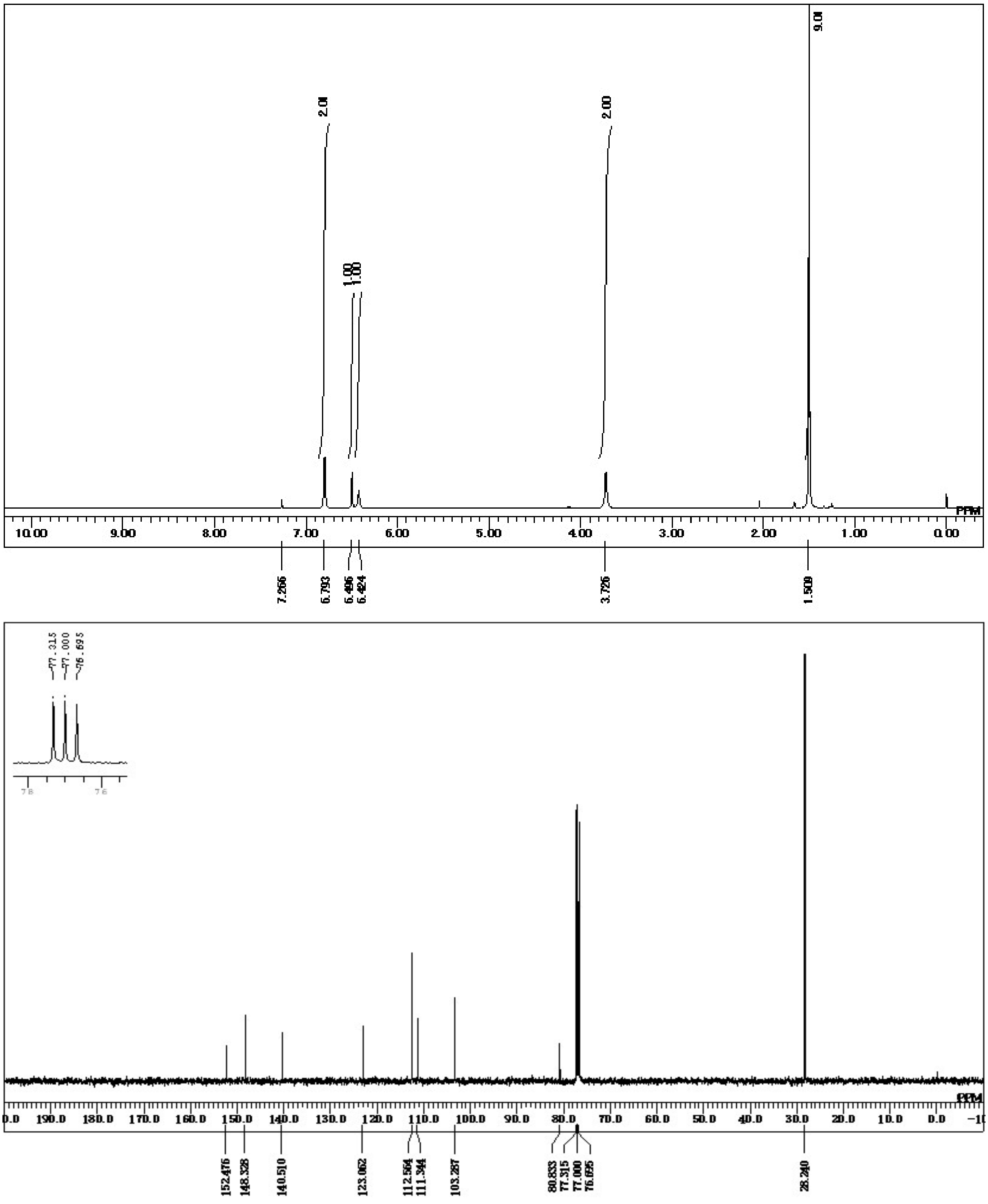

Figure S5. The NMR spectra of tert-butyl (3-amino-5-bromophenyl)carbamate (**6**). ^{1}H NMR (400 MHz, $CDCl_3$): δ 1.51 (9H, s), 3.73 (2H, s), 6.42 (1H, s), 6.50 (1H, s), 6.79 (2H, s). ^{13}C NMR (100 MHz, $CDCl_3$): δ 28.2, 80.8, 103.3, 111.3, 112.6, 123.1, 140.5, 148.3, 152.5. HRMS (FAB+) calculated for $C_{11}H_{15}BrN_2O_2$ [M+ H]$^+$: *m/z* = 286.0300, found 286.0317.

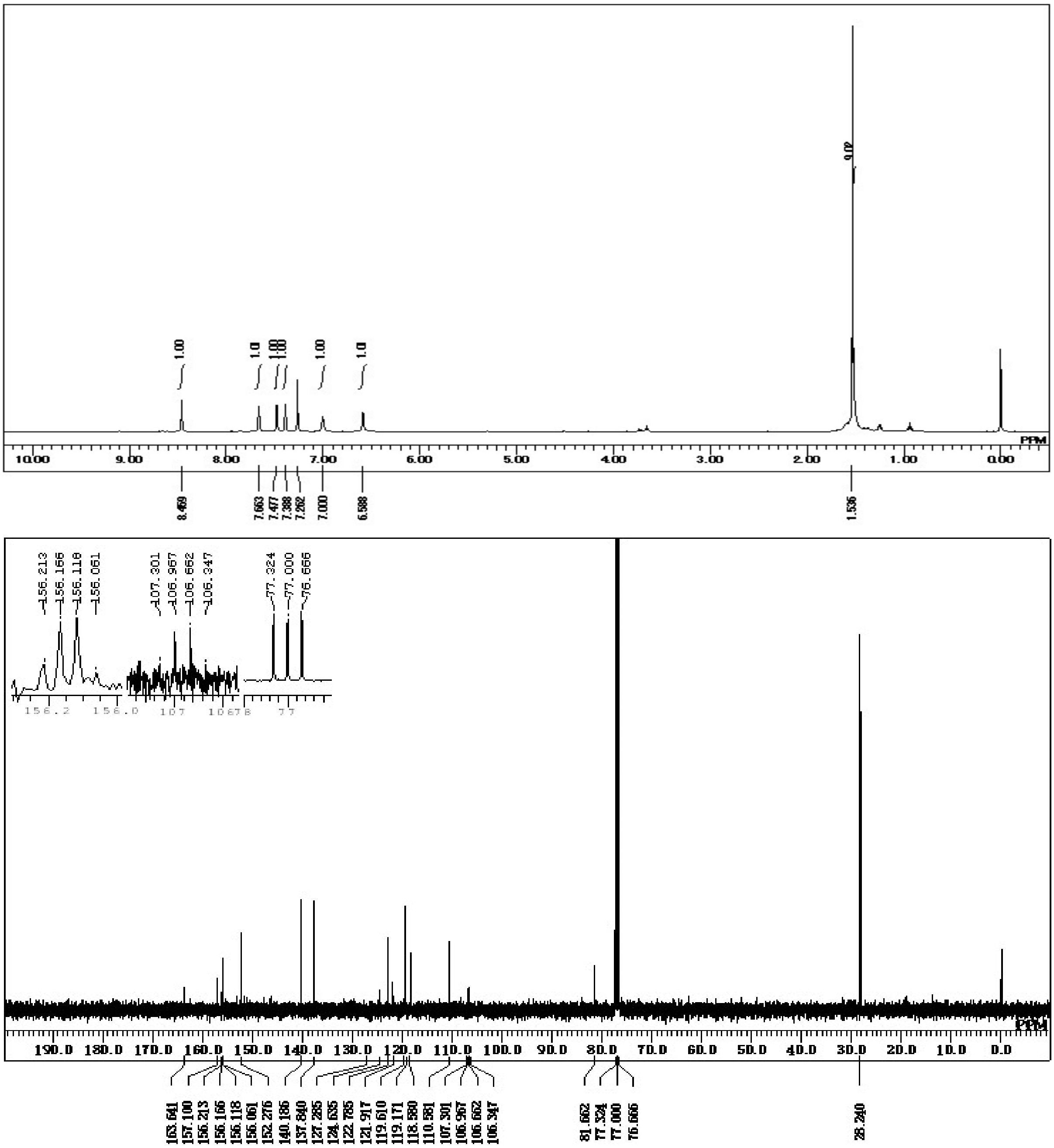

Figure S6. The NMR spectra of tert-butyl(3-{[2-chloro-5-(trifluoromethyl)pyrimidin-4-yl]amino}-5- bromophenyl)carbamate (**7**). ^{1}H NMR (400 MHz, $CDCl_3$): δ 1.54 (9H, s), 6.59 (1H, s), 7.00 (1H, s), 7.39 (1H, s), 7.48 (1H, s), 7.66 (1H, s), 8.46 (1H, s). ^{13}C NMR (100 MHz, $CDCl_3$): δ 28.2, 81.7, 106.8 (q, J_{CF} = 30.5 Hz), 110.6, 118.6, 119.6, 122.8, 123.4 (q, J_{CF} = 271.8 Hz), 137.8, 140.2, 152.3, 156.1 (q, J_{CF} = 4.8 Hz), 157.1, 163.6. HRMS (FAB+) calculated for $C_{16}H_{15}BrClF_3N_4O_2$ $[M+ H]^+$: *m/z* = 465.9854, found 465.9842.

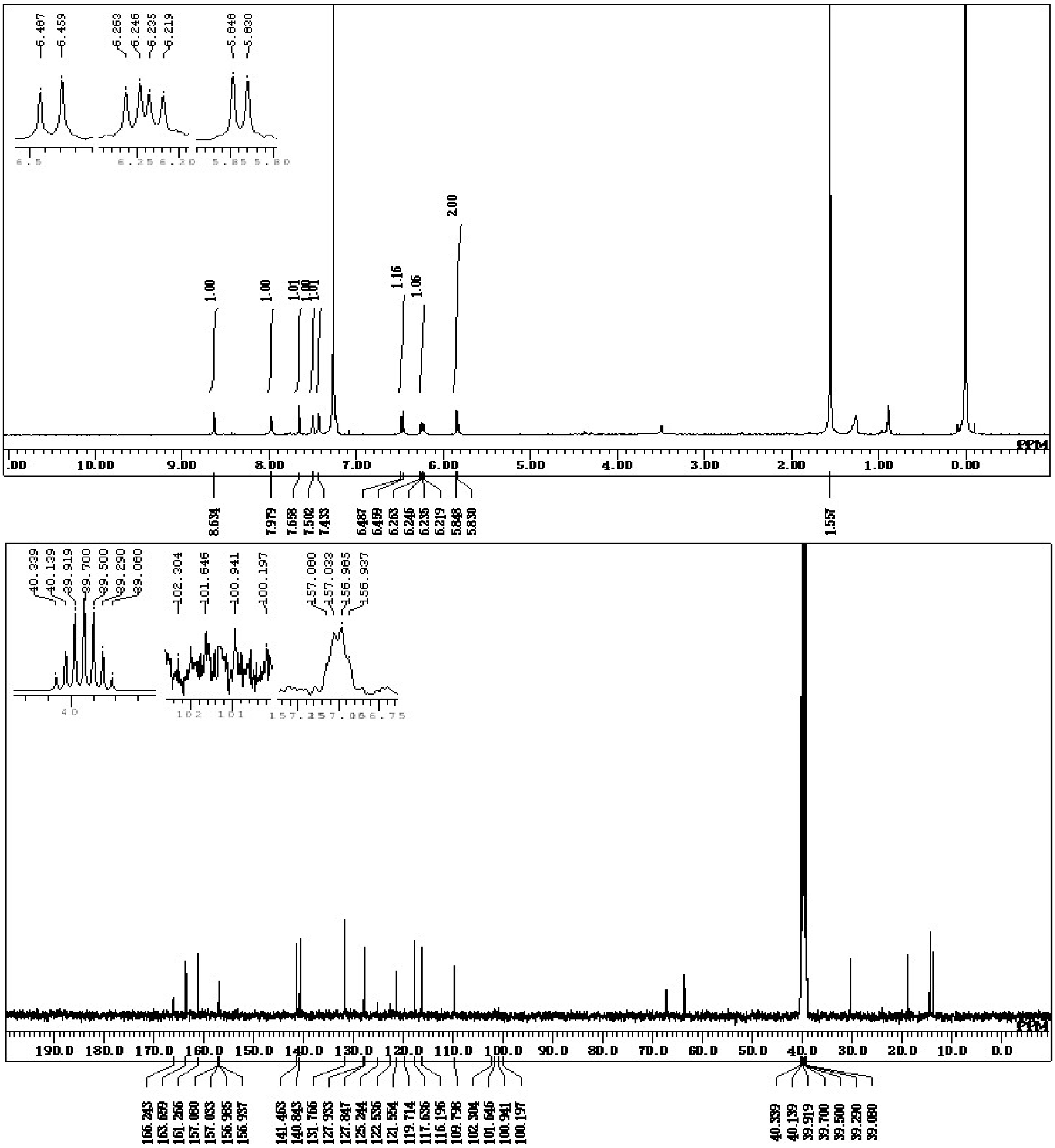

Figure S7. The NMR spectra of *N*-(3-{[2-chloro-5-(trifluoromethyl)pyrimidin-4-yl]amino}-5- bromophenyl)acrylamide (**8**). ^{1}H NMR (400 MHz, $CDCl_3$): δ 5.84 (2H, d, *J* = 7.2 Hz), 6.24 (1H, dd, *J* = 11.2, 6.8 Hz), 6.47 (1H, d, *J* = 11.2 Hz), 7.43 (1H, s), 7.50 (1H, s), 7.66 (1H, s), 7.98 (1H, s), 8.63 (1H, s). ^{13}C NMR (100 MHz, $(CD_3)_2SO$): δ 101.3 (q, J_{CF} = 70.5 Hz), 109.8, 116.2, 117.6, 121.6, 123.9 (q, J_{CF} = 270.8 Hz), 127.8, 131.8, 140.8, 141.5, 157.0 (q, J_{CF} = 4.8 Hz), 161.3, 163.7, 166.2. HRMS (FAB+) calculated for $C_{14}H_9BrClF_3N_4O$ $[M+H]^+$: *m/z* = 419.9702, found 419.9600.

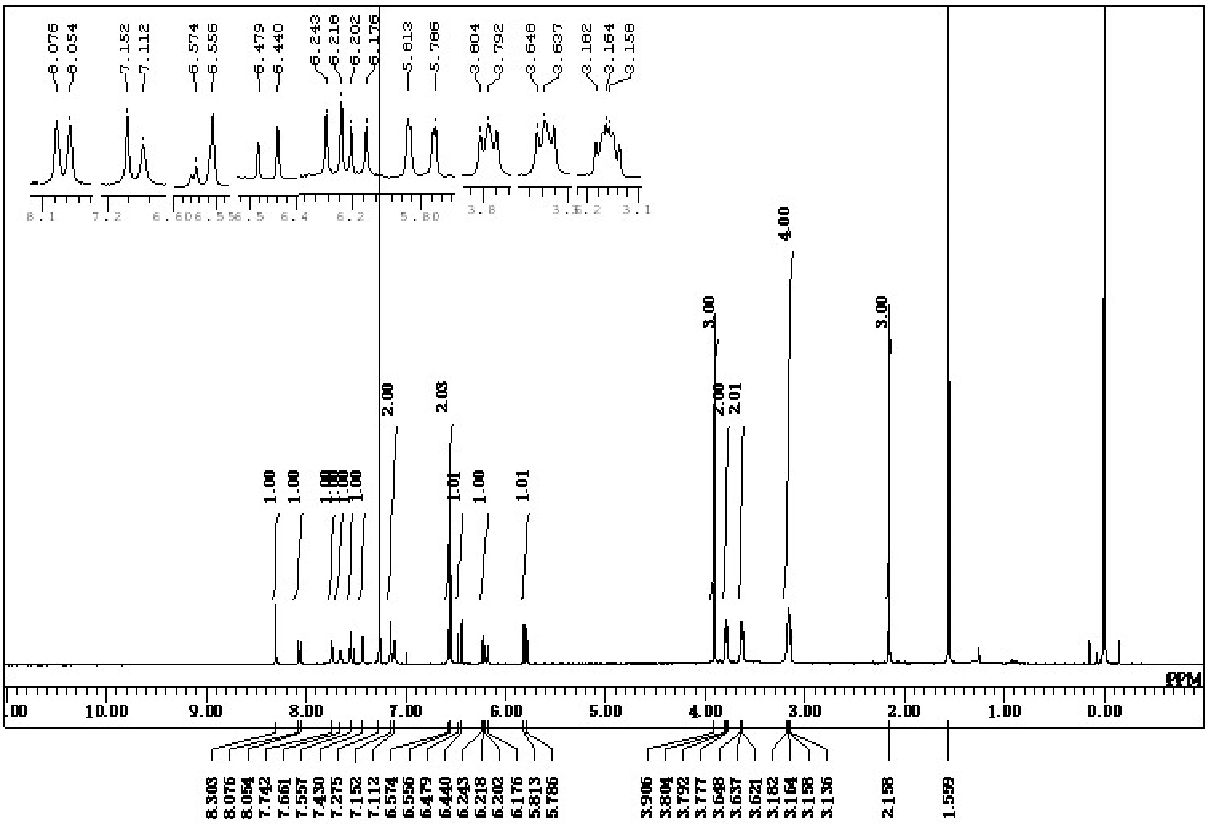

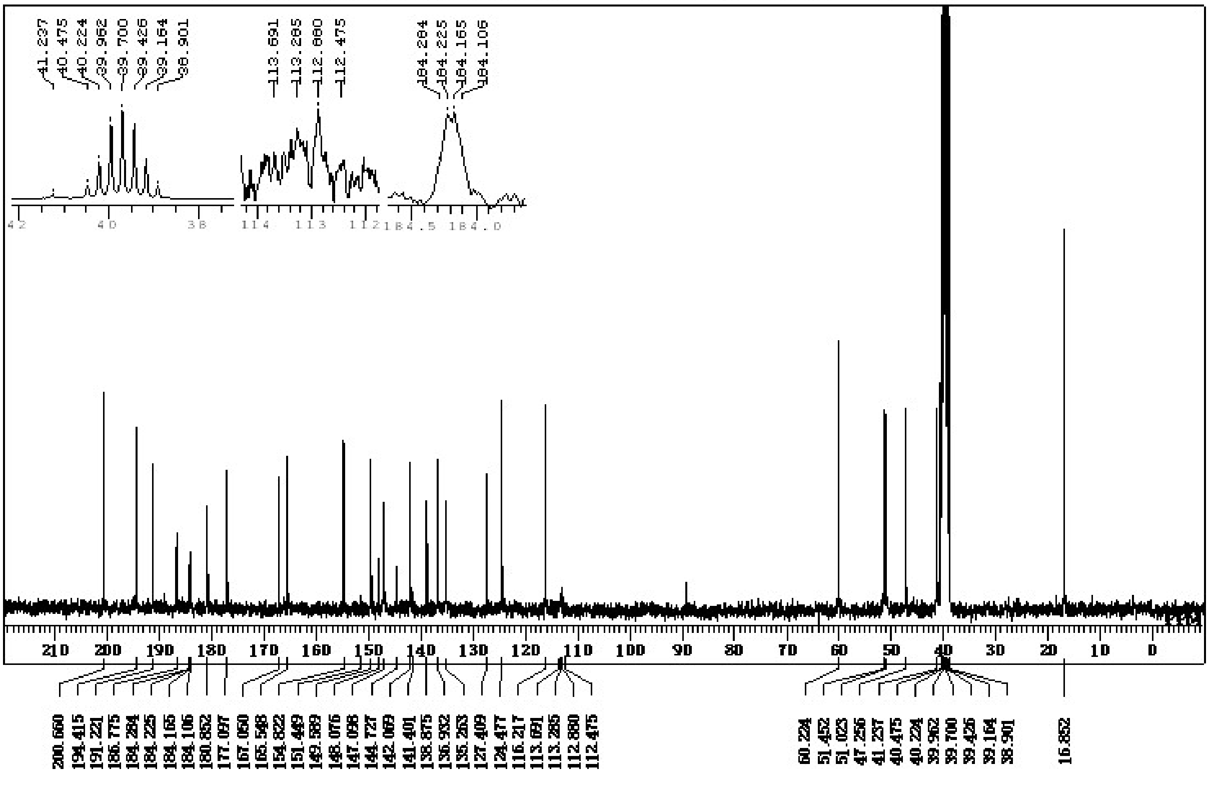

Figure S8. The NMR spectra of *N*-(3-{[2-({4-[4-acetylpiperazin-1-yl]-2-methoxyphenyl}amino)-5-(trifluoromethyl)pyrimidin-4-yl]amino}-5-bromophenyl)acrylamide (**9**). ^{1}H NMR (400 MHz, $CDCl_3$): δ 2.16 (3H, s), 3.12–3.20

(4H, m), 3.64 (2H, t, J = 4.4 Hz), 3.79 (2H, t, J = 4.8 Hz), 3.91 (3H, s), 5.80 (1H, d, J = 10.8 Hz), 6.21 (1H, dd, J = 15.2, 10.4 Hz), 6.44 (1H, d, J = 15.6 Hz), 6.54–6.60 (2H, m), 7.11 (1H, s), 7.25 (1H, s), 7.43 (1H, s), 7.56 (1H, s), 7.66 (1H, s), 7.74 (1H, s), 8.07 (1H, d, J = 8.8 Hz), 8.30 (1H, s). ^{13}C NMR (100 MHz, $(CD_3)_2SO$): δ 16.9, 41.2, 47.3, 51.0, 51.5, 60.2, 113.0 (q, J_{CF} = 40.5 Hz), 116.2, 124.5, 127.4, 135.3, 136.9, 138.9, 142.1, 146.4 (q, J_{CF} = 334.9 Hz), 147.1, 149.6, 154.8, 165.5, 167.1, 177.1, 180.9, 184.2 (q, J_{CF} = 6.0 Hz), 186.8, 191.2, 194.4, 200.7. HRMS (FAB+) calculated for $C_{27}H_{27}BrF_3N_7O_3$ [M+ H]$^+$: m/z = 633.1324, found 633.1311.
